# Microbial metabolite-mediated gut–liver axis remodeling after hepatectomy: mechanisms and therapeutic opportunities

**DOI:** 10.3389/fsurg.2026.1951535

**Published:** 2026-10-19

**Authors:** Chao Ji, Jing Zhang, Yan Jiao

**Affiliations:** 1 Department of The First Operation Room, The First Hospital of Jilin University, Changchun, Jilin, China; 2 Department of Hepatobiliary and Pancreatic Surgery, General Surgery Center, The First Hospital of Jilin University, Changchun, Jilin, China

**Keywords:** bile acids, gut–liver axis, hepatectomy, indole metabolites, intestinal barrier, liver regeneration, microbial metabolites, microbiota-directed therapy

## Abstract

Hepatectomy remains a cornerstone treatment for primary and metastatic liver malignancies, but successful recovery depends on coordinated liver regeneration, immune regulation, metabolic adaptation, and maintenance of intestinal homeostasis. Emerging evidence indicates that the gut–liver axis functions as a critical communication network mediated not only by microbial composition but also by microbial-derived metabolites. Surgical stress, perioperative fasting, antibiotic exposure, ischemia–reperfusion injury, altered portal circulation, and changes in bile acid metabolism can profoundly disrupt intestinal microbial ecology and reshape metabolite production after hepatectomy. This review will summarize current knowledge regarding microbial metabolites, including short-chain fatty acids, bile acid derivatives, indole metabolites, and other bioactive molecules, as regulators of liver regeneration and postoperative recovery. Particular attention will be given to their effects on intestinal barrier integrity, Kupffer cell activation, inflammatory signaling, mitochondrial metabolism, and extracellular matrix remodeling. We will further discuss emerging therapeutic strategies targeting the gut–liver metabolic axis, including nutritional modulation, probiotics, prebiotics, synbiotics, metabolite-based interventions, and microbiota-directed therapies. This review aims to provide a translational perspective on how microbial metabolites may serve as biomarkers and therapeutic targets for optimizing recovery after hepatectomy and improving metabolic resilience in patients undergoing liver surgery.

## Highlights

After hepatectomy, microbial metabolites help determine whether gut–liver signaling supports regeneration or amplifies postoperative injury. Short-chain fatty acids, microbial bile acid derivatives, indole metabolites, ketone bodies, and other bioactive signals can influence intestinal barrier integrity, Kupffer cell activation, IL-6/STAT3 signaling, mitochondrial adaptation, and extracellular matrix remodeling. Probiotics and synbiotics have the comparatively strongest clinical evidence, mainly for infection prevention, but heterogeneous regimens and patient populations preclude a class-wide claim that they improve liver regeneration; metabolite-based and microbiota-directed therapies remain investigational.

## Introduction

Hepatectomy is used for hepatocellular carcinoma, intrahepatic cholangiocarcinoma, colorectal liver metastases, and selected benign or premalignant conditions. The operation is safer than it used to be because imaging, volumetric planning, anesthesia, parenchymal transection, vascular control, and recovery pathways have improved. Even so, post-hepatectomy liver failure (PHLF) remains one of the complications surgeons fear most after major liver resection. The International Study Group of Liver Surgery defines PHLF as impaired synthetic, excretory, and detoxifying function after hepatectomy, reflected by increased international normalized ratio and hyperbilirubinemia on or after postoperative day 5 (1). Enhanced Recovery After Surgery (ERAS) recommendations for liver surgery address perioperative care, early mobilization, nutrition, and surgical stress (2, 3). They say much less about the microbial and metabolic signals that reach the liver through the portal circulation during the first days of regeneration.

Following partial hepatectomy, hepatocytes that had been in a quiescent state are triggered to re-enter the cell cycle, while non-parenchymal cells reshape the surrounding microenvironment—together enabling the liver to recover its mass without recapitulating the steps of embryogenesis. The classical framework divides this process into priming, proliferative, and termination phases, with contributions from tumor necrosis factor (TNF), interleukin-6 (IL-6), hepatocyte growth factor, epidermal growth factor receptor ligands, transforming growth factor beta, extracellular matrix reorganization, and metabolic reprogramming (4–6). Animal work has demonstrated that a lack of IL-6 leads to failed regeneration and overt liver failure (7), while intact TNF receptor 1 signaling proves essential for an appropriate regenerative response (8). Taken together, these observations underscore a key insight: inflammation, when properly regulated, is not merely a postoperative complication but an integral component of the regenerative process itself.

Given that the portal circulation drains the intestine directly, it is reasonable to consider the gut as a source of regenerative cues. Indeed, early work established that endotoxin of intestinal origin could stimulate the secretion of hepatotrophic factors during liver regeneration (9). Later studies implicated Toll-like receptor (TLR)/myeloid differentiation factor 88 (MyD88) signaling in murine liver regeneration (10), although MyD88-dependent cytokine production may occur independently of CD14, TLR2, and TLR4 in some models (11). The TLR4–MyD88 axis has further been connected to IL-6 activation in the aftermath of partial hepatectomy (12). Under normal conditions, microbial-associated molecular patterns function as priming stimuli; however, when the intestinal barrier is compromised or microbial exposure becomes excessive, the very same conduit can deliver harmful inflammatory insults. Bile acids represent yet another channel of gut-liver communication.

Once a portion of liver has been removed, the remaining tissue faces an altered bile acid flux. While bile acid signaling is indispensable for regeneration, an overload of bile acids carries the risk of damaging both hepatocytes and cholangiocytes. Nuclear receptor-mediated bile acid signaling has been shown to be necessary for normal regenerative outcomes (13), and bile salts exert regulatory control over hepatocyte proliferation, bile acid biosynthesis, and enterohepatic feedback loops (14, 15). The Farnesoid X receptor (FXR) and Takeda G protein-coupled receptor 5 (TGR5) pathways serve to align regenerative activity with bile acid homeostasis, shielding the remnant liver from bile acid toxicity while simultaneously facilitating metabolic adjustment (16–18). Because the conversion of primary to secondary bile acids depends on gut microbes, post-hepatectomy bile acid dynamics are inseparable from the microbiota. Conceptually, the gut–liver axis offers a useful organizing principle in this context, as it brings several postoperative phenomena into a shared framework—namely, microbial community structure, barrier integrity, immune-mediated signaling, bile acid handling, and microbially derived metabolites.

The wider liver-disease literature characterizes the gut–liver axis as a bidirectional network linking intestinal permeability, dysbiosis, microbial products, and hepatic inflammatory responses (19, 20). Microbiota–bile acid interactions and microbial metabolite signaling to distant organs are well established (21, 22), and the liver–microbiome axis is increasingly recognized as a regulator of hepatic immunity and metabolism (23). For clinicians managing patients after hepatectomy, the practical question is how to preserve beneficial metabolite-mediated signals while limiting bacterial translocation, infection, and inflammatory injury. Accordingly, this review organizes current evidence around microbial metabolites as regulators, biomarkers, and potential therapeutic targets during postoperative recovery.

## Literature search and scope

To compile this narrative review, we conducted systematic searches of PubMed and CrossRef using terms encompassing partial hepatectomy, liver regeneration, gut microbiota, intestinal barrier dysfunction, bacterial translocation, short-chain fatty acids, bile acids, indole metabolites, microbial metabolites, probiotics, synbiotics, prebiotics, fecal microbiota transplantation (FMT), and perioperative nutrition. In selecting sources, priority was given to mechanistic investigations in partial hepatectomy models, clinical studies of liver resection or broader hepatobiliary surgery, existing surgical guidelines, and current reviews of the gut–liver axis. Given the heterogeneity of the evidence and the largely experimental nature of many studies, this review emphasizes mechanistic integration rather than a formal meta-analytic synthesis.

## Conceptual model: metabolite-mediated signaling from regeneration to injury

In the post-hepatectomy setting, gut–liver signaling lies on a spectrum from regulated portal exposure to pathological bacterial translocation. Physiological exposure to microbial products and metabolites helps maintain hepatic immune tone and can prime Kupffer cells, activate IL-6 and TNF cascades, and facilitate hepatocyte cell-cycle entry. Major surgery disrupts this equilibrium. As summarized in Figure 1, intestinal hypoperfusion, fasting, opioids, antibiotics, bowel edema, cholestasis, ischemia–reperfusion injury, and stress hormones can alter metabolite production, microbial compartmentalization, and tight junction integrity. When the barrier fails, the same portal route can deliver excessive lipopolysaccharide, bacterial DNA, extracellular vesicles, and pro-inflammatory metabolites to a liver remnant already facing substantial metabolic demand.

**Figure 1:**
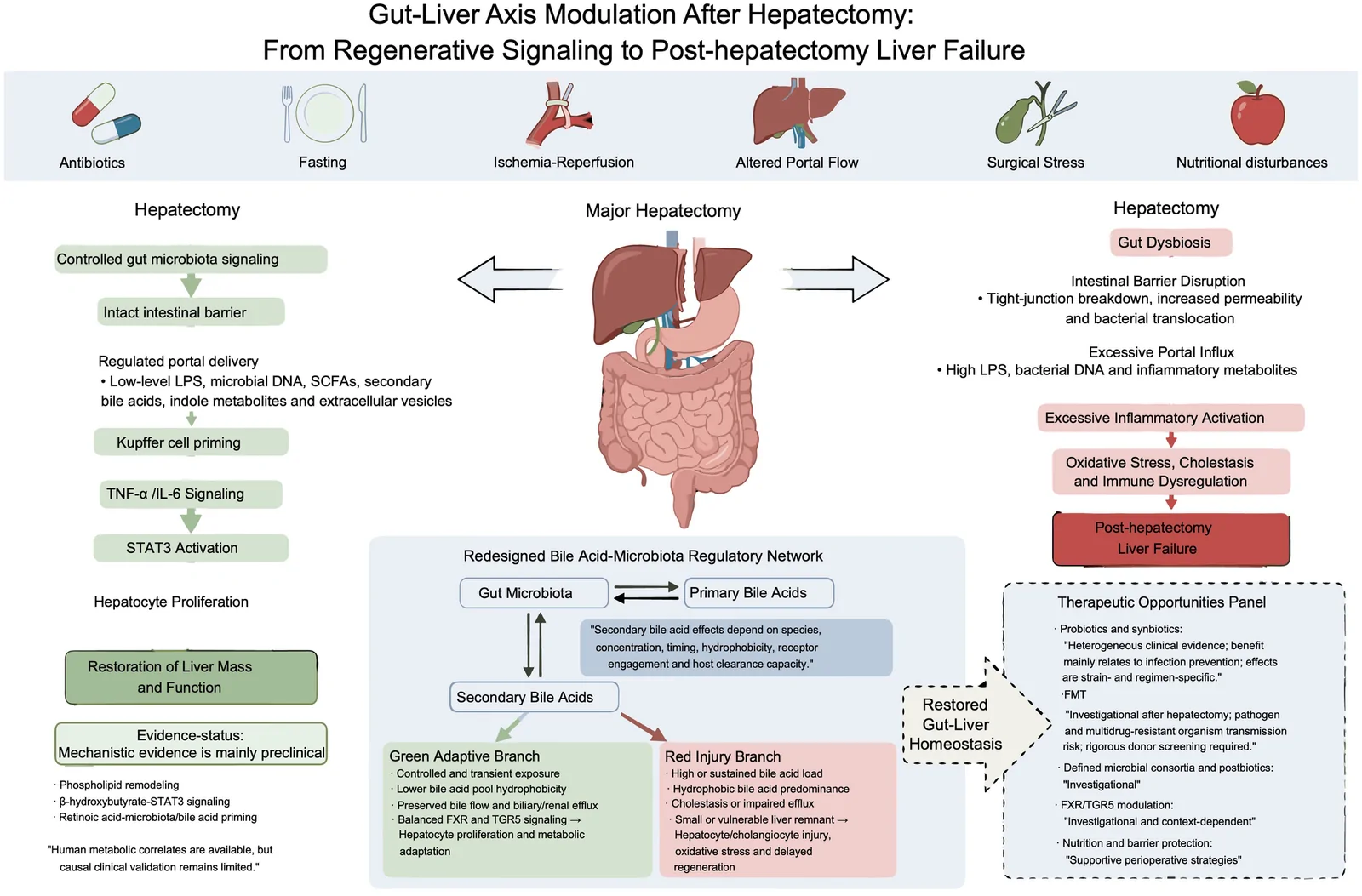
Metabolite-mediated remodeling of the gut–liver axis after hepatectomy: from regenerative signaling to post-hepatectomy liver failure. Following major hepatectomy, antibiotics, fasting, ischemia–reperfusion injury, altered portal blood flow, surgical stress, and nutritional disturbances reshape microbial ecology and metabolite production. Under physiological conditions, preservation of intestinal barrier integrity permits regulated portal delivery of microbial-associated molecular patterns and metabolites, including low-level lipopolysaccharide (LPS), microbial DNA, short-chain fatty acids (SCFAs), secondary bile acids, indole metabolites, β-hydroxybutyrate, and extracellular vesicles. These signals can modulate Kupffer cells, tumor necrosis factor-alpha (TNF-α), interleukin-6 (IL-6), and signal transducer and activator of transcription 3 (STAT3), while influencing mitochondrial metabolism, extracellular matrix remodeling, hepatocyte proliferation, and restoration of liver function. Conversely, dysbiosis and barrier disruption promote bacterial translocation and excessive portal influx of inflammatory signals, contributing to oxidative stress, cholestasis, immune dysregulation, and post-hepatectomy liver failure (PHLF). The bile acid–microbiota network further modulates regeneration through primary and secondary bile acids and downstream farnesoid X receptor (FXR) and Takeda G protein-coupled receptor 5 (TGR5) signaling. Candidate interventions, most of which remain investigational after hepatectomy, include nutritional modulation, probiotics, synbiotics, prebiotics, postbiotics, metabolite-based therapies, FMT, bile acid receptor modulation, and barrier protection.

As recent reviews on microbiota-derived signals in liver injury and regeneration have highlighted, this reflects a genuinely dualistic role: microbial cues are capable of supporting regenerative processes, yet dysbiosis and barrier breakdown can exacerbate both sterile and infectious postoperative complications (24, 25). Both the composition of the microbiota and the bile acid profile have been shown to shape the trajectory of liver injury and regeneration (26). In experimental partial hepatectomy, the intestinal microbiome has been linked to hepatic gene networks governing metabolism, cell-cycle regulation, and immune signaling (27). Retinoic acid pretreatment was found to hasten regeneration, an effect accompanied by shifts in both the microbiota and bile acid landscape (28). On the other hand, antibiotic-mediated disruption of commensal bacteria compromised regeneration by undermining innate immune tolerance in the liver (29). Observations from both human and animal studies further connect alterations in gut microbial communities with the pace and quality of liver regeneration (30). Collectively, these findings argue strongly against blanket gut sterilization as a therapeutic approach. A more rational objective would be to modulate signaling in a controlled, targeted fashion.

## Microbial metabolite-mediated regulation of liver regeneration

Perhaps the most compelling support for a regenerative contribution of the gut microbiota comes from partial hepatectomy experiments in which liver mass recovery was altered by antibiotic administration, germ-free conditions, microbial recolonization, or defined shifts in community composition. A mechanistic study in *Journal of Hepatology* reported that gut microbiota promote liver regeneration through hepatic membrane phospholipid biosynthesis, linking microbial signals to the lipid remodeling needed by proliferating hepatocytes (31). This matters because cell-cycle entry after hepatectomy requires more than cytokines; hepatocytes also need membrane synthesis, mitochondrial adaptation, and redistribution of metabolic substrates.

Gut microbiota may matter most in marginal livers. In fatty liver disease models, microbiome and metabolomic analyses showed that gut microbiota influence liver regeneration, suggesting that baseline metabolic disease changes the regenerative signal received through the gut–liver axis (32). Gut recolonization after partial hepatectomy can alter hepatic matrisome gene expression, which suggests an effect on extracellular matrix organization as well as hepatocyte proliferation (33). This is clinically relevant because many patients who undergo liver resection have steatosis, steatohepatitis, diabetes, chemotherapy-associated liver injury, or cirrhosis.

### Short-chain fatty acids

Short-chain fatty acids (SCFAs), particularly acetate, propionate, and butyrate, connect microbial fermentation with colonocyte energy supply, tight junction maintenance, immune tolerance, and hepatic substrate metabolism (21, 22, 34). Perioperative fasting, low fermentable-substrate availability, and antibiotic exposure may therefore reduce both SCFA production and the ecological functions that sustain the intestinal barrier. Direct evidence that SCFA supplementation accelerates liver regeneration after hepatectomy remains limited, so SCFAs are best viewed at present as candidate mediators and biomarkers rather than established regenerative treatments.

### Indole metabolites and amino acid-derived signals

Microbial metabolism of tryptophan generates indole derivatives that can support epithelial barrier programs and tune mucosal immune signaling through receptors such as the aryl hydrocarbon receptor and pregnane X receptor. Their relevance after hepatectomy is biologically plausible because barrier integrity and portal immune tone shape the regenerative environment, but direct resection-specific evidence is sparse (22, 34). Postoperative studies should therefore measure indole metabolites alongside dietary tryptophan exposure, antibiotic use, barrier markers, and liver function rather than infer metabolite activity from microbial composition alone.

### Other bioactive mediators

Several taxa and metabolites have been proposed as mediators. *Parabacteroides distasonis* was reported to promote liver regeneration by increasing beta-hydroxybutyric acid production and beta-hydroxybutyrate-driven STAT3 signaling (35). Gut microbiota and metabolome analyses after two-thirds partial hepatectomy identified steroid hormone biosynthesis as a regeneration-related pathway (36). Secondary bile acids in portal blood, including deoxycholic acid, have been shown to contribute to hepatocyte proliferation in a rat model of partial hepatectomy (37). Together, these studies show that microbial signals act through specific metabolic, receptor, and transcriptional pathways rather than fitting a simple anti-inflammatory vs. pro-inflammatory binary.

Multi-omics approaches have made this mechanistic complexity more actionable. Integrated microbiota, metabolomic, and proteomic analyses after hepatectomy have identified responses spanning amino acid catabolism, lipid remodeling, mitochondrial metabolism, and inflammatory regulation (38). In mice subjected to 50% partial hepatectomy followed by acute liver injury, the intestinal microbiota shifted dynamically as injury resolved and regeneration progressed (39). Microbial extracellular vesicles may provide another communication channel: after liver surgery, microbiota transfer was associated with extracellular-vesicle migration and altered hepatic immunity (40). Liver microbial DNA signatures and extracellular-vesicle epitopes have also been proposed as candidate predictors of PHLF (41). Although preliminary, these findings support patient-specific biomarker strategies rather than uniform supplementation.

Translation of these mechanisms to humans remains incomplete. Longitudinal metabolomics in living liver donors has documented postoperative changes in phospholipid and bile acid metabolism, and a recent study found increased serum phosphatidylcholine 16:0/20:4 in patients after hepatectomy and proliferative activity in human liver organoids, providing a human correlate for phospholipid remodeling (42, 43). Human observations also show perioperative rises in beta-hydroxybutyrate in patients with impaired liver function (44), but no clinical study has established a beta-hydroxybutyrate-STAT3 causal axis. We found no direct clinical validation of retinoic acid-mediated microbiota/bile acid priming. These pathways should therefore be viewed as preclinical mechanisms with limited human metabolic correlates, not established therapeutic targets for human liver regeneration.

## Intestinal barrier dysfunction and bacterial translocation after hepatectomy

The intestinal barrier controls what reaches the liver through the portal vein. Classic animal studies showed bacterial translocation, intestinal ultrastructural injury, and increased cell membrane or capillary permeability early after major liver resection (45, 46). Intravascular volume support with dextran 70 reduced enteric bacterial translocation after partial hepatectomy in rats (47), and water-soluble ethylhydroxyethyl cellulose prevented bacterial translocation induced by major liver resection (48). These interventions are not current microbiome therapies, but the studies showed that hemodynamic and luminal conditions can decide whether bacteria or bacterial products cross the gut barrier after hepatectomy.

Tight junction disruption is one mechanism behind this process. Partial hepatectomy changed ileal tight junctions in rats (49). In porcine models, antioxidant treatment attenuated intestinal mucosal damage and gut barrier dysfunction after major hepatectomy (50), and bioartificial liver support reduced intestinal mucosal injury after major hepatectomy (51). In patients undergoing resection of liver metastases, low venous pressure strategies were associated with compromised enteric blood flow, and insulin-like growth factor binding protein-1 was proposed as a biomarker of intestinal barrier injury (52). Radiofrequency-assisted parenchymal resection also caused gut barrier disruption in a porcine model (53). Thus, barrier dysfunction is multifactorial, reflecting operative technique, hemodynamic shifts, and stress on the liver remnant, and it can secondarily reshape portal metabolite exposure.

In a rat model, splenic artery ligation lessened bacterial translocation after major hepatic resection (54), while N-acetylcysteine improved intestinal barrier integrity in partially hepatectomized animals (55). These studies suggest that reducing oxidative stress, limiting portal hyperperfusion, or improving mucosal perfusion could help preserve the gut–liver axis. Yet aggressive antimicrobial decontamination may also remove commensal-derived regenerative signals. Microbes in cecal crypts contribute to intestinal epithelial repair after surgical stress (56), and overnight fasting plus antibiotics can disrupt spatial organization of the cecal microbiome in mice (57). Perioperative care should therefore protect barrier function and metabolite-producing ecological niches without attempting wholesale microbial eradication.

## Kupffer cell and inflammatory signaling by gut-derived cues

Gut-derived ligands reach the liver through the portal vein and encounter Kupffer cells, liver sinusoidal endothelial cells, dendritic cells, hepatic stellate cells, and recruited innate immune cells. Physiological exposure to microbial-associated molecular patterns can induce TNF and IL-6, activate nuclear factor kappaB and STAT3, and prime hepatocytes for subsequent growth-factor stimulation. Microbial metabolites modify this response: SCFAs, secondary bile acids, indole derivatives, and ketone bodies may alter Kupffer cell activation, inflammatory thresholds, and hepatocyte mitochondrial metabolism through receptor-mediated and transcriptional pathways (21, 22, 24, 26, 35, 37). After major hepatectomy, however, barrier injury can increase portal exposure while detoxification capacity is reduced. Hepatic inflammation and cholestasis then disturb bile acid flow and microbial ecology, creating a cycle of dysbiosis and inflammatory metabolite production.

Notably, the IL-6/STAT3 pathway that underpins regeneration can, if activated excessively or for too long, also propagate systemic inflammation. Therapeutic strategies should therefore strive for immunologic calibration—fine-tuning the immune response—rather than indiscriminate suppression. This nuance has direct implications for the selection of antibiotics vs. probiotics. Although broad-spectrum antibiotics may curtail bacterial translocation in certain scenarios, they carry the risk of undermining the commensal-dependent immune tolerance that supports regeneration.

Probiotics and synbiotics may help prevent infection and reinforce barrier integrity, but their effects depend on strain, timing, and host risk. Patients with biliary obstruction, cirrhosis, prolonged antibiotic exposure, malnutrition, diabetes, or chemotherapy-associated liver injury may have distinct baseline microbiota, metabolite profiles, and immune tone. Trials should stratify for these factors rather than assuming that all hepatectomy patients share a uniform gut–liver state.

## Microbial bile acid derivatives after liver resection

Bile acids serve multiple biological roles—they act as detergents, metabolic hormones, antimicrobial agents, and substrates for microbial transformation. In the wake of hepatectomy, the processes governing bile acid synthesis, hepatic uptake, biliary efflux, and enterohepatic recirculation must all adapt rapidly to accommodate the reduced liver mass. FXR activation, occurring in both the liver and the intestine, downregulates bile acid production via the small heterodimer partner and fibroblast growth factor 15/19 cascades, with additional effects on lipid and glucose handling. Through TGR5, bile acids can modulate energy expenditure, inflammatory tone, and the threshold for bile acid tolerance. The gut microbiota, for its part, deconjugates and dehydroxylates bile acids, thereby shifting the balance between primary and secondary species and influencing receptor activation patterns.

The opposing effects of bile acids are determined by the total bile acid load and timing after resection; the hydrophobicity, conjugation state, and relative abundance of individual species; receptor engagement through FXR and TGR5; and host clearance capacity, including remnant volume, cholestasis, and biliary or renal efflux. In rodents, deoxycholic acid in portal blood contributes to hepatocyte proliferation (37), whereas TGR5 deficiency increases hydrophobic bile acid retention, necrosis, inflammation, and delayed regeneration (16). In 32 patients undergoing major hepatectomy, a more hydrophobic hepatic bile acid pool correlated with worse postoperative injury and cholestasis (58). A secondary bile acid therefore cannot be classified as uniformly beneficial or toxic: low, transient receptor-mediated signaling in an adequately draining remnant may be adaptive, whereas high or sustained exposure to hydrophobic species in cholestasis or impaired efflux is injurious. Bile acid-directed therapy after hepatectomy should therefore focus on measured pool composition and receptor activity rather than indiscriminate supplementation or suppression.

The bile acid-microbiota axis can also be measured. Serial serum and fecal bile acid profiling, portal or peripheral metabolomics, fibroblast growth factor 19, C4 as a marker of bile acid synthesis, and microbiome-derived secondary bile acid signatures could help distinguish a pro-regenerative state from an injurious one. These endpoints may be useful in extended hepatectomy, cholestatic tumors, portal vein embolization, associating liver partition and portal vein ligation for staged hepatectomy, and steatotic liver disease.

## Therapeutic opportunities along the gut–liver metabolic axis

### Probiotics and synbiotics

Perioperative probiotic or synbiotic therapy is the gut–liver intervention with the most clinical experience in hepatobiliary surgery. In biliary cancer surgery, perioperative synbiotic treatment reduced postoperative infectious complications in a randomized controlled trial (59). A randomized, double-blind pilot study of liver resection found that prebiotics and probiotics influenced postoperative recovery and liver regeneration endpoints, although the sample size was too small for firm conclusions (60). In hepatic surgery with or without cirrhosis, perioperative synbiotics affected infectious complications, intestinal integrity, fecal flora, and organic acids (61). Reviews of highly invasive abdominal surgery also support synbiotics for reducing infectious complications in selected settings (62).

A 2025 systematic review and meta-analysis pooled 19 randomized trials involving 1,698 patients across liver resection and transplantation and found fewer postoperative infections with perioperative probiotics or synbiotics (63). This aggregate estimate should not be interpreted as evidence for a uniform class effect. Formulations, strains, doses, treatment timing and duration, concomitant antibiotics, surgical populations, and outcome definitions varied across studies; many trials were small and measured infection rather than liver regeneration. A placebo-controlled trial in patients with chronic liver disease undergoing liver resection found no reduction in endotoxemia, postoperative liver dysfunction, or overall complications (64). Related transplantation trials reported fewer infectious complications in some cohorts (65–67), but transplantation is biologically and clinically distinct from liver resection. We therefore regard this evidence as comparatively strongest for infection prevention at the class level, while evidence for any specific regimen or a direct regenerative effect remains insufficient.

A synbiotic protocol effective in biliary cancer surgery may not translate to chemotherapy-treated colorectal liver metastases or cirrhosis. Safety is particularly important in immunosuppressed, critically ill, or central-line-dependent patients. Future trials should report infectious complications, PHLF using standardized definitions, bilirubin and international normalized ratio kinetics, liver volume recovery, inflammatory markers, intestinal permeability, microbiome composition, bile acid profiles, and microbial metabolites.

### Prebiotics, dietary modulation, and perioperative nutrition

Compared with direct microbial supplementation, prebiotics and dietary modification may represent a more physiologically grounded approach, insofar as they reshape the ecological niche rather than simply introducing selected organisms. Fermentable fiber substrates can boost the production of short-chain fatty acids (SCFAs)—notably acetate, propionate, and butyrate—each of which plays a role in modulating epithelial barrier integrity, immune tolerance, and hepatic metabolic function. Sufficient protein intake is equally important, given that liver regeneration draws on amino acid pools for *de novo* protein synthesis, nucleotide biosynthesis, and immune-mediated tissue repair. Extended periods of fasting, pre-existing malnutrition, sarcopenia, and excessive catabolism in the perioperative period can all undermine both barrier function and the liver’s capacity to regenerate.

Current ESPEN recommendations for clinical nutrition in liver disease advise routine screening for malnutrition along with the provision of adequate caloric and protein intake, with particular emphasis on patients with cirrhosis or advanced hepatic dysfunction (68, 69). Within the specific context of liver surgery, nutritional status, nutritional supplementation, the gut microbiota, ischemia-reperfusion injury, and regenerative outcomes are all closely intertwined (34, 70, 71). A nutrition strategy oriented around the gut–liver axis in the post-hepatectomy period should, where clinically appropriate, incorporate early enteral feeding, generous protein provision, minimization of unnecessary fasting periods, glycemic control, and—in patients without ileus or other contraindications—consideration of fiber or prebiotic supplementation. Caloric adequacy remains important, but the health of the intestinal barrier and the stability of the microbial ecosystem deserve equal attention.

### Fecal microbiota transplantation and defined microbial consortia

FMT can restore microbial diversity and metabolic function more broadly than a single probiotic strain, but this theoretical advantage does not establish perioperative benefit. Direct evidence after hepatectomy is lacking. Clinical experience in liver disease comes mainly from cirrhosis, hepatic encephalopathy, alcohol-associated liver disease, and metabolic liver disease. A randomized trial showed that FMT from a rational stool donor improved hepatic encephalopathy in cirrhosis (72), and the THEMATIC trial further evaluated microbiota transplant for hepatic encephalopathy (73). In severe alcohol-associated hepatitis, FMT has been compared with prednisolone in a randomized trial (74). These studies support feasibility in selected liver-disease populations but do not establish safety or efficacy after liver resection.

For hepatectomy, FMT should remain investigational and should not be proposed for routine perioperative use. Risks include transmission of enteric pathogens and antimicrobial-resistance genes, donor-derived bacteremia or sepsis, aspiration or procedure-related complications, and uncertain interactions with antitumor immunity or immunosuppressive therapy. Genomically linked extended-spectrum beta-lactamase-producing E. coli bacteremia occurred in two immunocompromised FMT recipients, one of whom died (75). Contemporary guidance therefore emphasizes rigorous donor history, testing for multidrug-resistant organisms and emerging pathogens, product traceability, informed consent, and post-treatment surveillance (76). Routine clinical use is centered on recurrent or refractory Clostridioides difficile infection; use in postoperative, transplant, cancer, or other liver-disease populations remains investigational. The altered barrier and immune state immediately after hepatectomy may increase risk, and direct safety data in this setting are absent. Defined microbial consortia or postbiotic products may offer more standardized and controllable alternatives to whole-stool transplantation.

### Metabolite-based interventions

Direct metabolite-based interventions could reduce the variability of live microbial products, but evidence after hepatectomy is currently preclinical. Candidate approaches include SCFA- or indole-derived postbiotics, beta-hydroxybutyrate-related strategies, and enzymes or substrates that reshape secondary bile acid production (34, 35, 37). Their effects are likely to depend on dose, route, timing, cholestasis, residual liver volume, and baseline microbial ecology. Early studies should pair safety and pharmacokinetic measures with barrier, immune, mitochondrial, and regeneration endpoints rather than assume that a metabolite beneficial in chronic liver disease will also be beneficial immediately after resection.

### Barrier-protective and bile acid-targeted strategies

Barrier protection may be as relevant as microbial composition. Measures that preserve intestinal perfusion, avoid excessive venous pressure reduction, reduce oxidative stress, maintain enteral nutrition, and limit unnecessary broad-spectrum antibiotics may lower bacterial translocation. Pharmacological agents such as antioxidants, bile acid receptor modulators, and compounds that reinforce tight junction integrity remain at an experimental stage in the hepatectomy setting, yet the biological rationale for their use is sound.

Strategies targeting bile acids, however, will require particularly careful development. FXR agonists, TGR5 modulators, bile acid sequestrants, ursodeoxycholic acid, and microbiota-guided bile acid remodeling interventions may each have distinct effects depending on the presence or absence of cholestasis, the size of the liver remnant, and the composition of the gut microbiota. Given that bile acid signaling is a prerequisite for successful regeneration, indiscriminate suppression of this pathway carries the potential for harm. Future trials should therefore incorporate direct measurements of bile acid pools and receptor-associated biomarkers, rather than relying solely on conventional liver enzyme values as surrogate endpoints.

## Biomarkers and patient stratification

The next step for gut–liver axis research in hepatectomy is to progress beyond correlative associations toward risk-based patient stratification. A range of candidate biomarkers has emerged, including fecal microbial diversity, the relative abundance of bile acid-transforming taxa, plasma lipopolysaccharide-binding protein, soluble CD14, endotoxin activity assays, intestinal fatty acid binding protein, zonulin-related markers, citrulline levels, SCFAs, indole derivatives, individual bile acid species, microbial extracellular vesicle epitopes, bacterial DNA signatures, and standard liver function tests. Imaging-based assessments of liver volume recovery, together with functional measures such as indocyanine green clearance, could be integrated with microbiome and metabolomic datasets to more reliably differentiate true regeneration failure from transient cholestasis or generalized systemic inflammation.

The patient subgroups most likely to derive benefit from stratified gut–liver interventions include those undergoing major hepatectomy, individuals with a small future liver remnant, and those with cholestatic injury, cirrhosis, steatotic liver disease, diabetes, sarcopenia, prolonged preoperative antibiotic exposure, biliary drainage, neoadjuvant chemotherapy, or elevated infection risk. Across these groups, baseline microbiota composition, bile acid pool characteristics, immune tone, and regenerative reserve can vary substantially. Applying a uniform perioperative microbiota protocol to all comers risks diluting any potential benefit while obscuring signals of harm.

## Trial design priorities

A critical priority for future clinical trials is the disaggregation of endpoints that have traditionally been lumped together—specifically, infection prevention, barrier protection, metabolic support, and liver regeneration. Infection-related endpoints should encompass surgical site infections, cholangitis, intra-abdominal abscess formation, bacteremia, duration of antibiotic therapy, and length of hospital stay. Barrier-related endpoints ought to include permeability assessments, markers of intestinal injury, and indicators of bacterial translocation. Metabolic endpoints should capture bile acid composition, SCFA levels, amino acid profiles, ketone body concentrations, and patterns of lipid remodeling. Regeneration endpoints should incorporate PHLF grading, the kinetics of bilirubin and coagulation recovery, liver volume restoration, functional liver testing, and serum markers of regenerative activity.

The temporal dimension of intervention deserves careful attention. Prehabilitation may be needed to address dysbiosis, malnutrition, and sarcopenia before surgery. Perioperative strategies should preserve barrier integrity during fasting, anesthesia, and ischemia–reperfusion stress, whereas postoperative interventions can support recovery after enteral nutrition resumes. These windows should not be conflated.

A probiotic initiated only after complications have already set in addresses a fundamentally different clinical question than a synbiotic administered for one to two weeks preoperatively. In addition, trials must incorporate mechanistic sampling protocols. Reliance on stool samples alone is insufficient, given that the relevant signals reach the liver via the portal circulation and may be more faithfully reflected in bile acid profiles, circulating metabolites, extracellular vesicles, and immune mediator levels. Where practically and ethically feasible, studies should collect paired preoperative and postoperative stool and blood specimens, bile samples when surgical drains are in place, and clinical data that are temporally aligned with liver function trajectories.

## Limitations of current evidence

Several caveats warrant a cautious interpretation of the evidence reviewed here. A substantial proportion of the mechanistic data derives from rodent partial hepatectomy models in which genetic background, diet, microbiota composition, and resection volume are tightly controlled—conditions that do not reflect the variability seen in clinical practice. Patients undergoing hepatectomy differ widely with respect to tumor biology, underlying liver parenchyma, prior chemotherapy exposure, the presence of biliary obstruction, portal hypertension, and the specifics of perioperative management. Moreover, microbiome studies frequently establish associations without demonstrating causation, and fecal samples may not adequately capture the mucosa-associated or small intestinal microbial communities that are most relevant to gut-liver interactions. Many of the probiotic and synbiotic trials conducted to date have been small in scale, have employed heterogeneous formulations, and have focused primarily on infectious complications rather than on direct measures of liver regeneration. It is also conceivable that microbiota modulation could cause harm to certain patients, especially if it raises the risk of bacteremia or conflicts with essential antimicrobial treatment. Lastly, the therapeutic window for inflammatory and bile acid signaling after hepatectomy is likely to be narrow; overly aggressive suppression of danger signals may, paradoxically, hinder the regenerative process.

## Conclusion

Microbial metabolite-mediated gut–liver signaling is a credible regulator of recovery after hepatectomy. SCFAs, microbial bile acid derivatives, indole metabolites, ketone bodies, extracellular vesicles, and other bioactive signals may connect intestinal barrier integrity with Kupffer cell activation, inflammatory priming, mitochondrial adaptation, extracellular matrix remodeling, and hepatocyte proliferation. Surgical stress can shift this network from regulated portal communication toward dysbiosis, bacterial translocation, infection, cholestasis, and PHLF. Probiotics and synbiotics have the comparatively strongest clinical evidence, mainly for reducing postoperative infection, but heterogeneity in formulations, dosing, timing, patient populations, and endpoints limits regimen-specific conclusions and does not establish a direct regenerative effect. Direct metabolite therapies, FMT, defined consortia, postbiotics, and bile acid-directed approaches remain investigational. Progress will require risk-stratified perioperative studies that measure microbial ecology and metabolite exposure together with barrier, immune, metabolic, and regeneration endpoints. Until such evidence is available, microbial metabolites should be regarded as promising biomarkers and therapeutic targets rather than established regenerative treatments.
